# Kushenin combined with entecavir for chronic hepatitis B: A protocol for a systematic review and meta-analysis

**DOI:** 10.1097/MD.0000000000018898

**Published:** 2020-01-31

**Authors:** Yunxia Wang, Jiayuan Zhang, Yuxia Jin, Xiao Xiao, Qi Zhang

**Affiliations:** Chengdu University of Traditional Chinese Medicine, Chengdu, Sichuan Province, China.

**Keywords:** chronic hepatitis B, entecavir, kushenin, meta-analysis, oxymatrine, protocol

## Abstract

**Background:**

A recent study has reported that there are >240 million patients infected with chronic hepatitis B (CHB) worldwide. Once patients with CHB start antiviral treatment, they need to take antiviral drugs for a long period, which may lead to a series of side effects, and the resistance to the antiviral drugs may also emerge. We aim to evaluate the efficacy and safety of kushenin (KS) combined with entecavir (ETV) for chronic hepatitis B.

**Methods:**

Randomized controlled trials (RCTs) of KS combined with ETV for CHB will be identified from PubMed, EMBASE, Web of Science, The Cochrane Library, Chinese Biomedical Database, China National Knowledge Infrastructure, Chongqing VIP, Wangfang Data. Literature screening and data extraction will be independently performed by 2 researchers. The cochrane collaboration tool for assessing risk of bias will be applied to evaluate the risk of bias of the RCTs included. The extracted data will be analyzed by Rev-man 5.3.0 software.

**Results:**

A high-quality synthesis of current evidence on the efficacy and safety of KS combined with ETV for CHB will be provided in this study.

**Conclusion:**

This systematic review will aim to evaluate the efficacy and safety of KS combined with ETV for CHB.

**PROSPERO registration number:**

CRD42019124790.

## 1 Introduction

More than 240 million people worldwide are suffering from chronic hepatitis B (CHB).^[1]^ Once antiviral therapy is initiated, patients with CHB need to take antiviral drugs for a long period of time, which may lead to related side effects and drug resistance. The mechanism of nucleos (t)ide analogues in the treatment of CHB is to inhibit viral replication and reduce serum Hepatitis B virus DNA (HBV-DNA) titer. However, its effect on reducing hepatitis B virus surface antigen (HBsAg) transcription is very limited.^[2]^ Entecavir (ETV) is one of the preferred antiviral agents for antiviral therapy recommended in several guidelines.^[3,4]^ Kushinin (KS) is an active alkaloid extracted from *Sophora flavescens*, of which the main component is oxymatrine. KS has been proved to have antiviral, immunomodulatory, and anti-inflammatory effects,^[5]^ several studies^[6,7]^ have shown that KS combined with other antiviral drugs have a better antiviral effect in reducing the level of serum virological indicators, improving drug resistance and delaying the progression of liver fibrosis. This meta-analysis aims to provide a basis for clinical treatment for CHB, and explore whether KS combined with ETV has the advantages of improving efficacy and reducing side effects in antiviral therapy for patients with CHB.

## 2 Materials and methods

### 2.1 Registration

The protocol of this systematic review has been registered in PROSPERO (Registration Number: CRD42019124790).

### 2.2 Inclusion criteria

#### 2.2.1 Types of studies

The randomized controlled clinical trials (RCTs) of KS combined with ETV for CHB will be included.

#### 2.2.2 Types of patients

Patients with the diagnosis of CHB^[8]^: positive hepatitis B virus surface antigen and/or positive serum HBV-DNA for more than half a year, elevated serum alanine aminotransferase (ALT) level, there is no limitation on the age, sex. Patients who have coinfection with other types of hepatitis virus, HIV infection, autoimmune hepatitis, drug-induced liver injury, hereditary liver disease, hepatic carcinoma, and so on will be excluded.

#### 2.2.3 Types of interventions

The experimental group should be treated with KS combined with ETV, with no restriction on dosage form, including oral administration, intravenous drip or acupoint injection, while the control group should be treated with ETV alone. The course of treatment should be >3 months, and if there were other treatments, the 2 groups should be consistent.

#### 2.2.4 Types of outcomes

The main outcomes are the effective rate of serum virological indicators and hepatic function indicator. Additional outcomes are the effective rate of serum hepatic fibrosis indicators, drug resistance rate, side effects rate.

### 2.3 Searching strategy

Literature searching will cover both English and Chinese electronic databases, including PubMed, EMBASE, Web of Science, The Cochrane Library, Chinese Biomedical Database, China National Knowledge Infrastructure, Chongqing VIP, Wangfang Data. Medical subject headings combine with text word searching will be performed, search terms include Hepatitis B, Chronic, CHB, entecavir, baraclude, kushenin, oxymatrine, matrine. The date ranges from the establishment of databases to February 2019.

### 2.4 Data collection and analysis

#### 2.4.1 Selection of studies and data extraction

**Figure 1:**
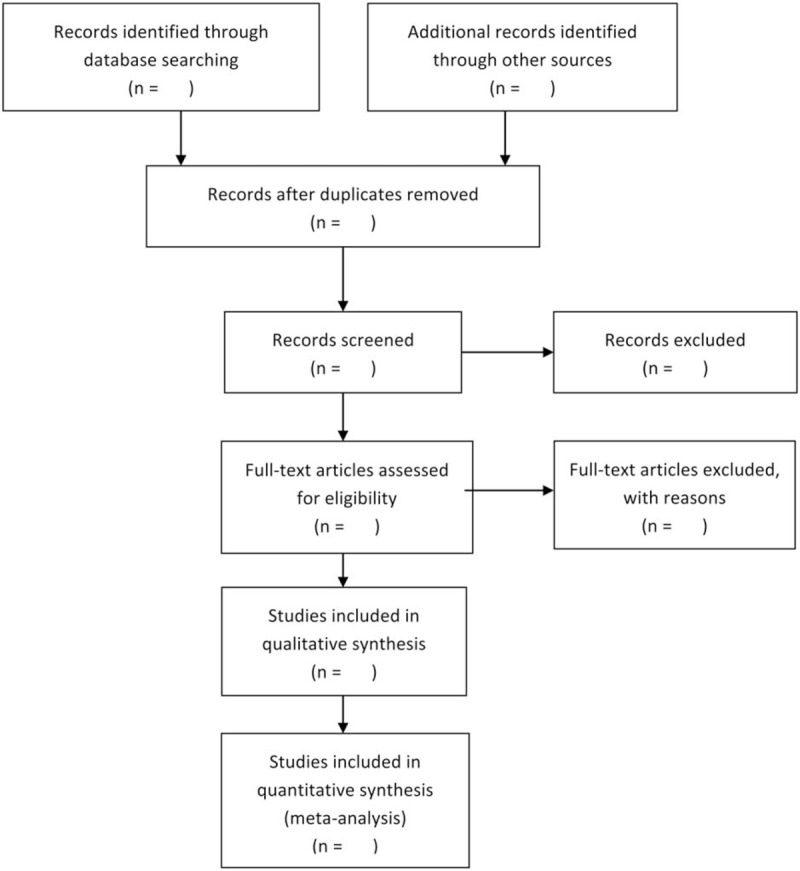
Flow of literature screening.

Literature screening and data extraction will be carried out independently by 2 researchers (YW, JZ) using the unified scale, and cross-checking will be conducted. The inclusion of controversial literature will be determined by a third researcher (QZ). If the required information is incomplete, we will contact the author to obtain the necessary data. The entire process is performed in the flow diagram (Fig. 1).

#### 2.4.2 Assessment of risk of bias

The cochrane collaboration tool for assessing risk of bias will be applied to evaluate the risk of bias of the RCTs included. The scale contains 7 parts, including random sequence generation (selection bias), allocation concealment (selection bias), blinding of participants and personnel (performance bias), blinding of outcome assessment (detection bias), incomplete outcome data (attrition bias), and selective outcome reporting (reporting bias). The judgment is categorized as “Low risk” of bias, “High risk” of bias, or “Unclear risk” of bias.

#### 2.4.3 Measures of treatment effect

The extracted data will be analyzed using the Rev-man 5.3.0 (Copenhagen, Denmark) software provided by the cochrane collaboration. For continuous variable, mean difference (MD) and 95% confidence intervals (95% CI) will be reported. And for dichotomous variable, risk ratio (RR) and 95% CI will be reported.

#### 2.4.4 Assessment of heterogeneity and data synthesis

Statistical heterogeneity will be assessed by *Q*-statistic and *I*^2^ statistic. If there is no significant heterogeneity (*P* > .10, *I*^2^ < 50%), the data will be assessed by fixed-effects model. Otherwise, subgroup analysis and sensitivity analysis will be performed to identify the potential source of heterogeneity, and the data will be assessed by random effects model.

#### 2.4.5 Assessment of reporting bias

Publication bias will be assessed by a funnel plot when the included studies is >10.

#### 2.4.6 Subgroup analysis and sensitivity analysis

Subgroup analysis will be conducted if there is a significant heterogeneity (grouped by treatment duration, dosage form, sample size) and sensitivity analysis will be performed to identify the potential source of heterogeneity.

#### 2.4.7 Confidence in cumulative evidence

GRADE system will be used for assessing the strength of the body of evidence,^[9]^ of which the quality of evidence will be categorized as high quality, moderate quality, low quality, and very low quality.

## 3 Discussion

The possible antiviral mechanism of KS are interfering with the packaging of HBV paired-guide RNA nucleocapsid, and inhibiting the activity of viral DNA polymerase.^[10]^ It was also been suggested^[11]^ that KS can inhibits the expression of heat-stress cognate 70, a host protein required for HBV replication. In a study published in 2017,^[12]^ ETV showed a better efficacy than KS in reducing HBV replication and serum HBV-DNA level, while KS was more effective in reducing serum HBsAg, hepatitis B virus e antigen (HBeAg), and hepatitis B virus c antigen (HBcAg) level, which may explain the improved antiviral effect of KS combined with ETV. This systematic review aims to evaluate the efficacy and safety of KS combined with ETV for CHB. In order to provide more references for the clinical treatment of CHB.

## Author contributions

None.
